# Graves' Disease and the Post-partum Period: An Intriguing Relationship

**DOI:** 10.3389/fendo.2019.00853

**Published:** 2019-12-10

**Authors:** Laura Croce, Giulia Di Dalmazi, Francesca Orsolini, Camilla Virili, Giulia Brigante, Elena Gianetti, Mariacarla Moleti, Giorgio Napolitano, Massimo Tonacchera, Mario Rotondi

**Affiliations:** ^1^Unit of Internal Medicine and Endocrinology, Laboratory for Endocrine Disruptors, Istituti Clinici Scientifici Maugeri IRCCS, Pavia, Italy; ^2^PHD course in Experimental Medicine, University of Pavia, Pavia, Italy; ^3^Unit of Endocrinology, Department of Medicine and Sciences of Aging, “G. D'Annunzio” University of Chieti-Pescara, Chieti, Italy; ^4^Endocrinology Section, Department of Clinical and Experimental Medicine, University of Pisa, Pisa, Italy; ^5^Endocrinology Unit, Department of Medico-Surgical Sciences and Biotechnologies, Sapienza University of Rome, Latina, Italy; ^6^Unit of Endocrinology, Department of Biomedical, Metabolic and Neural Sciences, University of Modena and Reggio Emilia, Modena, Italy; ^7^Department of Clinical and Experimental Medicine, University of Messina, Messina, Italy; ^8^Department of Internal Medicine and Therapeutics, University of Pavia, Pavia, Italy

**Keywords:** thyroid, Graves' disease, post-partum, pregnancy, iodine deficiency, antithyroid drugs, lactation, autoimmunity

## Abstract

The post-partum period is an immunologically peculiar period in a woman's life. Indeed, most of the pregnancy-related immune changes gradually revert in the 12 months following delivery. Although the post-partum period has long been identified as a period of aggravation of autoimmune thyroid diseases, most of the currently available studies took into account the relationship between post-partum and autoimmune thyroiditis. More recently, the potential repercussions of the post-partum period on Graves' disease were also taken into account. The present mini review will briefly overview the most recent advances in our knowledge of the immunology of the post-partum period in relation with the potential repercussions on the clinical course of Graves' disease. Moreover, some peculiar aspects of post-partum Graves' disease in terms of clinical and biochemical presentation, diagnostic challenges, and specific therapeutic considerations also taking into account the recommendation of the latest clinical guidelines on the management of thyroid diseases in pregnancy will be overviewed.

## Introduction

The post-partum (PP), at least for the potential repercussions on thyroid homeostasis, is generally defined as the 12 months after delivery. At difference with the mechanisms of immune deviation occurring during pregnancy that have been extensively studied, little has been done to investigate the immune profile of the PP and the related recovery of the immune system. Briefly, the PP period is characterized by an immune rebound from the pregnancy-related immunological deviation. It is well-known that the dynamic changes occurring in both cellular and humoral immunity may reflect the different timing of exacerbation or new onset of some autoimmune diseases, including autoimmune thyroid diseases (1). However, if the occurrence of post-partum thyroiditis (PPT), its clinical course, and peculiar aspects have been objects of several studies (2), the potential peculiarities of Graves' disease (GD) occurring and/or re-exacerbating in the PP period were by far less extensively characterized. The main aim of the present review will be to overview the current evidences addressing the issue of the association between PP and GD with particular regards to specific clinical aspects and therapeutic indications.

## Immunology of the PP Period

A successful pregnancy represents an immunological challenge for the maternal immune system since it has to defend both mother and fetus from foreign pathogens and in the meanwhile tolerance for paternal alloantigens expressed in fetal tissues must be ensured in order to prevent rejection of the fetus. Hence, the mother's immune system undergoes profound changes during pregnancy (3).

As a consequence of the Th1–Th2 immune shift that occurred during gestation, the PP period is characterized by an immunological rebound toward a Th1 immune-phenotype, which was demonstrated to be associated with a worsening of specific autoimmune diseases and/or with an exacerbation of quiescent or latent infections (4). The timing and degree of this “immune reconstitution” has not been fully clarified, but at least from a thyroidal point of view, most of the pregnancy-induced immune changes gradually return to normal within 12 months from delivery (5).

The immune changes occurring in the PP period involve both the innate immune system and the adaptive one. Watanabe et al. (6), reported that, in healthy women, an early increase of helper and cytotoxic T cells and a delayed increase of suppressor and TCR alpha beta-negative T cells occurs in the PP. More recently, a recovery of the majority of B cell subsets was also demonstrated in the PP period (7). The same group has shown that serum markers of B cells activation, such as soluble CD23 and B-cell-activating factor (BAFF), and IgG levels gradually increased during the PP period (8). Interestingly, both regulatory T- and B-cells, essential to control maternal immune tolerance toward fetus in early pregnancy, were found to be increased during the PP period compared to third trimester and day of delivery (7, 9). This latter finding highlights a dynamic behavior of these cells' subsets and the changes occurring after parturition.

Moreover, profound changes in inflammatory and anti-inflammatory molecules characterize the pregnancy to PP transition, as recently reported by Brann et al. (10) who found a decrease of the major anti-inflammatory markers in the PP period.

## Is the PP Period a High Risk Moment for *de novo* Occurrence of GD?

Early studies addressing the issue of *de novo* GD development in the PP period consistently reported that a relevant percentage of childbearing aged women with GD showed a PP onset of the disease (11–13). According to Benhaim Rochester and Davies, as much as 45% of GD diagnosed in parous women in childbearing age showed a PP occurrence and the estimated relative risk for PP-GD peaked at 5.6 for the age group 35–39 years when compared to the control population (13). However, these previous findings were not confirmed by a more recent Italian study specifically addressing the issue. (14). Indeed, the positive predictive value of the PP period for the onset of GD was <10%, thus supporting the concept that the role of the PP period as a major risk factor for *de novo* occurrence of GD had been somehow overestimated. Furthermore, the stratification of childbearing aged patients according to a PP or a non-PP onset of GD allowed establishing that those with a PP onset of GD were characterized by younger age and were more likely to have a positive family history for AITD, as compared with those showing a non-PP onset of GD. These results would fit with the concept that PP would act as a precipitating event for the onset of GD in genetically predisposed women, rather than as a causative factor. Several considerations may be helpful for explaining the discrepancy between studies addressing this issue. Indeed, exclusion of patients in non-childbearing age in some studies (11, 13), the use of early TRAb assay methods, at least for the earliest studies (11, 12), and/or the lack of systematic thyroid scintiscan and/or ultrasound evaluation could have led to misclassification of patients with transient thyrotoxicosis due to PPT with the final result of strengthening the weight of the PP period as a risk factor for the onset of GD.

However, besides the above differences, it seems reasonable concluding that discrepant results between the two most recent studies were due to changes of the objectivity rather than by different quality of the studies evaluating the issue. Indeed, the progressive reduction in the parity rate that occurred, in Italy as well as in most developed countries, over the last two decades clearly affects the strength of the PP period as a risk factor for the onset of GD.

It should be highlighted that in the study by Rotondi et al. (14), a lower mean number of successful pregnancies as compared with the one by Benhaim Rochester and Davies (13) was observed.

### Is the PP Period a High Risk Moment for the Relapse of GD?

The medical treatment of Graves' hyperthyroidism with thionamides (methimazole or propylthiouracile), although effective in restoring euthyroidism, is associated with a high rate of relapsing hyperthyroidism once these drugs are discontinued (15, 16).

Pregnancy is known to be associated with a clinical remission/amelioration of GD, as assessed by a reduction of the dose and/or withdrawal of anti-thyroid drugs (ATD) during gestation. In particular, in the last trimester of pregnancy, discontinuation of ATD therapy is achieved in as much as 20–30% of patients with active GD (17, 18). On the other hand, several longitudinal studies demonstrated that the clinical course of GD re-exacerbates after delivery, as assessed by the need for re-introduction and/or dose increase of ATD in order to maintain euthyroidism (19).

Recent Japanese study showed that continuation of ATD throughout pregnancy would be associated with a reduction of the risk of PP recrudescence of Graves' hyperthyroidism, although this therapeutic option is not frequently feasible in view of the need to protect the fetus from excessive levels of ATD during gestation (20).

Besides women entering pregnancy while on ATD treatment for active GD, the role of PP period as a risk factor for relapsing Graves' hyperthyroidism appears strengthened if we take into account the findings obtained in women with GD in long-term remission after ATD withdrawal. There is only one study specifically comparing the relapse rate in patients with GD in euthyroidism after a full cycle of ATD therapy in relation to the occurrence or absence of a full-term pregnancy after the stopping of MMI (21). The authors reported that the risk of relapse was higher in patients who had at least one pregnancy after ATD withdrawal as compared to those who did not deliver following ATD withdrawal. Given that the vast majority of relapse occurs within the first 18 months after ATD withdrawal (22), the strikingly high relapse rate (84%) observed in long-term remitted patients strongly points toward a relevant role of PP period in driving GD relapse.

Based on the above findings, and at difference with the 2012 guidelines on thyroid dysfunctions during pregnancy that recommended to monitor thyroid function only in women with active GD (23), a suggestion regarding the monitoring of thyroid function in women with a history of remitted GD was given in the 2017 ATA guidelines (24).

## Peculiar Aspects of PP Relapses of GD

Potential peculiarities in the clinical phenotype, including severity of hyperthyroidism and TSH-receptor Ab (TRAb) levels at presentation, clinical response, and long-term outcome of PP relapsing Graves' hyperthyroidism, were not systematically evaluated. Benhaim Rochester and Davies first reported that patients with PP onset of GD showed a non-significant trend for a better response to ATD as compared with those with a non-PP onset (13). More recently, a specifically designed study compared the clinical and biochemical phenotype of patients with relapsing Graves' hyperthyroidism according to a PP and a non-PP (NPP) onset. The results demonstrated an overall identical phenotype as assessed by superimposable FT3, TRAb titers, thyroid volume, smoking habit, presence, and degree of orbitopathy at diagnosis of relapse independently of a PP or NPP onset (25). More importantly, despite similar starting dose of MMI and duration of treatment, patients with a PP relapse were characterized by a strikingly higher success rate of MMI as opposed to those with NPP relapse (79% vs. 32%). Unfortunately, no data were reported about the rate of patients requiring definitive treatment of hyperthyroidism. It could be reasonable to hypothesize that the transient immunologic rebound occurring after delivery (in particular the Th2 to Th1 return shift and a dysregulation of Treg cells) (1) played a role in determining recurrence while the restoration of the pre-pregnancy immune balance (3) would have favored achieving remission. To further strengthen the above statement, it seems worthwhile highlighting that TRAb levels significantly decreased after 12 months of MMI treatment in patients with a PP relapse but not in those with a NPP relapse. This could indicate that the above cited immune restoration phase might have synergized with the immune modulating effects of MMI, in reducing the circulating concentrations of TRAb (26–29). Based on the above findings, it would seem reasonable to recommend that patients experiencing a PP relapse of Graves' hyperthyroidism should be addressed to a second course of ATD treatment rather than to definitive treatment.

## Differential Diagnosis of Thyrotoxicosis in the PP Period

At difference with pregnancy, during which GD accounts for most cases of hyperthyroidism (30, 31), PP thyroiditis (PPT) is by far the most common etiology sustaining thyrotoxicosis in the PP period (32–35). Furthermore, while in early pregnancy the differential diagnosis is mainly performed between Graves' hyperthyroidism and gestational transient thyrotoxicosis (36, 37) in the PP period, the differential diagnosis should mainly take into account GD and the thyrotoxic phase of PPT.

Thyroid scintiscan may be useful as a tool for establishing the differential diagnosis between GD and PPT. While 131I use should be kept to a minimum during breastfeeding because of its long half-life, short half-life isotopes such as 123I as well as 99mTc could be considered, with the precaution of interrupting the breastfeeding for 24 h in case of 99mTc and 3–4 days in case of 123I (38). However, nowadays, the use of sensitive TRAb assay methods provides a sensitive and easily feasible diagnostic alternative (39). It should be remembered that the lack of systematic TRAb measurements in the past has likely played a role in over-estimating the prevalence of GD occurring in the PP period. Thyroid ultrasound findings may also be helpful; indeed, both lower thyroid volume and intrathyroidal blood characterize patients with PPT as compared to those with GD (40). In addition to these tools, there are several aspects that may support the clinicians in rendering the correct diagnosis. These include the following: (i) extrathyroidal manifestations are clearly diagnostic even if rarely present; (ii) the timing of onset of PP thyrotoxicosis, which is usually shorter for PPT than for GD (according to a Japanese study, 86% of patients showing thyrotoxicosis within 3 months after delivery had PPT, while the development of thyrotoxicosis later than 6.5 months after delivery was in all cases due to GD) (38); (iii) the duration of thyrotoxic phase is a further discriminating aspect, in that in patients with a thyrotoxicosis lasting more than 3 months, the diagnosis of PPT should be questioned (41); (iv) owing to the different physiopathology of the two clinical entities, higher FT3/FT4 ratio usually characterizes GD (42).

The striking differences of these two conditions must be recognized from the endocrinologist to decide the proper clinical management. In fact, no treatment may be required in PPT patients unless symptomatic treatment with β-blockers to dominate hyperkinetic cardiovascular activity. In these patients, antithyroid treatment is unuseful and even detrimental, while it clearly represents the first-line treatment for patients with GD (41).

## Iodine Supplementation in PP Period

Inadequate iodine intake during pregnancy has long been known to be associated with gestational goitrogenesis in the mother (43) and with impaired fetal brain development (44). More recently, it became clear that the increased iodine requirement does not end with delivery, but rather it persists in lactating women. In this latter situation, estimating the optimal iodine intake should take into account both maternal and neonatal requirements (45). The United States Institute of Medicine has set the Recommended Daily Allowance (RDA) for iodine during breastfeeding at 290 μg per day, which is an even higher amount than that recommended during gestation (46). It should be highlighted that a recent study demonstrated that, in countries with an effective salt iodization program, iodine supplementation for lactating mothers could be unnecessary (47). However, both the Endocrine Society and ATA Guidelines unanimously recommend ≈250 μg of dietary iodine daily to all breastfeeding women (23, 24). Although no specific recommendation for patients with GD is given in the above-cited guidelines, women with active or previous GD should not be discouraged to take iodine supplements during lactation, since breast milk is the only source of iodine for breast-fed infants and iodine supplementation at the usual doses has no impact on maternal hyperthyroidism.

Although recommendations on the need for iodine supplementation in moderately-to-severely iodine deficient areas are clear, an American epidemiologic study reported that the majority of obstetricians and midwives do not recommend iodine-containing supplements in women during lactation, thus reinforcing the necessity of more information programs for professionals (48).

## Safety of ATD in the PP Period and Lactation

Based on the demonstration that both methimazole (MMI) and propylthiouracil (PTU) are transferred into the breast milk, for many years women taking either of these drugs were advised by their physicians not to breast-feed in order to avoid the risk of hypothyroidism in the infant. At difference with these early indications, nowadays, there is general consensus that breastfeeding in patients treated with ATD for GD should by no way be discouraged (49). Indeed, several studies evaluating thyroid function in infants whose mothers breast-fed while taking PTU or MMI failed to detect adverse effects on the newborn. Accordingly, studies first performed using PTU confirmed that only a very small amount of the drug is transferred from maternal serum into breast milk, well-below a therapeutic dose and deemed to pose no risk to the thyroid function of breastfeeding infant. Kampmann et al. (50) reported that <0.1% of the administered dose of propylthiouracil entered maternal milk and that the maximum concentration of PTU in milk was only about 10% of the maximum concentration in serum. Consequently no changes in circulating thyroid function parameters could be observed in the baby of the PTU-treated mother.

Studies on MMI or CM confirmed a four- to seven-fold higher proportion of the medication transferred into maternal milk in comparison to PTU, because MMI is minimally bound to serum proteins, whereas PTU is more extensively protein-bound in serum, mostly to albumin. In addition, MMI has a high solubility and it is not ionized in serum whereas PTU is more ionized in serum (pH 7.4) than in the more acidic breast milk (pH 6.8), thus inhibiting its transfer from serum into the lipid-rich breast milk (51–53). Approximately 0.1–0.2% of an orally administered MMI/CM dose is excreted into breast milk.

In his studies, Azizi (54) demonstrated that treatment of hyperthyroidism with a daily MMI dose of 5–20 mg in lactating mothers has no effects on thyroid function of the breast-fed infants (55), even in those whose mothers had marked increase in serum TSH concentrations.

Finally, potential allergic effects associated with ATD therapy, such as rash, agranulocytosis, hepatic dysfunction, and autoimmune sequelae, have not been reported in infants who breast-fed while their mothers were treated with ATD. In summary, maternal ATD use during lactation appears to be safe, whether it is continued after gestation or initiated in the PP period (56).

The child's thyroid function does not need to be checked regularly as long as somatic and mental development progress normally and if the mother breast-feeds 2-h or more after PTU ingestion (57, 58).

Taken together, the above evidences supported the safety of low to moderate doses of both PTU and MMI/CM during breastfeeding (59). PTU may theoretically be preferred over MMI because of its lower milk/serum concentration ratio (0.1 vs. 1) (56). Momotani et al. (57) showed that the mother can breast-feed safely while taking PTU in doses as high as 750 mg daily and that there is not a significant correlation between PTU doses and infants' TSH concentrations. Accordingly, the 2017 ATA Guidelines (24) and the 2018 ETA Guidelines (60) recommend that the lowest effective dose of MMI/CM or PTU should be administered.

While both guidelines agree on the safety threshold for MMI (<20 mg/day), they differ with regard to PTU: ETA recommends a <250 mg/day dose (60), while ATA increased the threshold from 300 mg/day in 2011 (23) to 450 mg/day in the latest guideline (24).

Finally, ATD should be taken after having breastfed the child and in fractioned doses (24).

## Future Research Requirements in the Field

Many aspects regarding GD and the PP period still need to be clarified. Studies regarding the immunological perturbations occurring in women who experience PP onset or relapse of GD will help us elucidate the pathophysiological mechanism underlying this phenomenon. Moreover, clinical studies on large populations are needed to identify predictive factors for PP relapses and also to evaluate long-term clinical outcomes. Also, the issue of iodine supplementation during lactation in GD women has not been extensively studied up to now, and future studies will be needed to identify the correct timing and dosage of iodine supplementation in this subset of patients.

## Author Contributions

LC, GD, FO, CV, GB, and EG performed the literature review and wrote the first draft of manuscript. MM, GN, MT, and MR revised and edited the manuscript. All authors read and approved the submitted version.

### Conflict of Interest

The authors declare that the research was conducted in the absence of any commercial or financial relationships that could be construed as a potential conflict of interest.
